# Condylar Remodeling and Skeletal Changes Following Occlusal Splint and Manual Therapy: A Cone Beam Computed Tomography Study in Temporomandibular Disorders

**DOI:** 10.3390/jcm13185567

**Published:** 2024-09-19

**Authors:** Manuela Tăut, Ioan Barbur, Mihaela Hedeșiu, Alina Ban, Daniel Leucuța, Marius Negucioiu, Smaranda Dana Buduru, Aranka Ilea

**Affiliations:** 1 Department of Oral Rehabilitation, “Iuliu Hațieganu” University of Medicine and Pharmacy, 400012 Cluj-Napoca, Romania; manuela.taut@elearn.umfcluj.ro (M.T.);; 2 Department of Prosthetic Dentistry and Dental Materials, Prosthetic Dentistry, “Iuliu Hațieganu” University of Medicine and Pharmacy, 400006 Cluj-Napoca, Romania; 3 Department of Maxillo-Facial Surgery and Radiology, Surgery and Maxillo-Facial Implantology, “Iuliu Hațieganu” University of Medicine and Pharmacy, 400029 Cluj-Napoca, Romania; 4 Department of Maxillo-Facial Surgery and Radiology, Dental Radiology, “Iuliu Hațieganu” University of Medicine and Pharmacy, 400029 Cluj-Napoca, Romania; 5 Department of Medical Education, Medical Informatics and Biostatistics, “Iuliu Hațieganu” University of Medicine and Pharmacy, 400349 Cluj-Napoca, Romania

**Keywords:** biomedical imaging, cone beam computed tomography, musculoskeletal disorders, occlusal splints, physical therapy, therapeutic exercise

## Abstract

**Background**: Temporomandibular disorders (TMD) may be associated with degenerative disease of temporomandibular joint (TMJ), such as condyle erosion and subchondral cysts. Occlusal splint and cranio-mandibular manual therapy, or combined therapy, is recommended as a conservative treatment to alleviate pain-related signs and symptoms in TMD. This study aimed to assess osseous condylar changes and skeletal changes following occlusal splint and cranio-mandibular manual therapy in TMD using cone beam computed tomography (CBCT). **Methods**: A retrospective cohort study included 24 patients diagnosed with TMD. Combined therapy was performed until pain-related signs and symptoms disappeared. CBCT scans were performed before and after therapy. Osseous structure of condyles and their subsequent modifications were analyzed on CBCT images: flattening, erosion, and subchondral cyst. Sella-Nasion-A point (SNA), Sella-Nasion-B point (SNB), A point-Nasion-B point (ANB), Sella-Articulare-Gonion (Condylar angle), and anterior and posterior facial height (AFH, PFH) were measured on CBCT-generated lateral cephalograms. A paired *t*-test, Wilcoxon rank-sum test, McNemar test, and Stuart–Maxwell test were used for the statistical analyses. **Results**: The treatment period with combined therapy was 7.42 ± 3.27 months, and 21 out of 33 TMJ presenting degenerative disease (63.6%) had significant complete remodeling (*p* < 0.05). Following therapy, SNB significantly decreased from 75.61 ±3.47° to 74.82 ± 3.41° (*p* = 0.02), ANB significantly increased from 4.05° (3.35–4.9°) to 4.8° (3.3–6.12°) (*p* < 0.001), AFH significantly increased from 112.85 mm (109.28–118.72) to 115.3 mm (112.58–118.88) (*p* < 0.001), PFH/AFH significantly decreased from 64.17 (61.39–66.1) to 63 (59.68–64.51) (*p* = 0.012), and condylar angle significantly increased from 140.84 ± 8.18° to 144.42 ± 8.87° (*p* = 0.007). **Conclusion**: Combined therapy promoted significant condylar remodeling in TMJ degenerative disease, along with skeletal changes (mandibular retrusion and increase in facial height). Therapeutic strategies should consider condylar remodeling in TMD. Skeletal and dental parameters should be evaluated prior to occlusal splint therapy.

## 1. Introduction

Temporomandibular disorders (TMD) are defined as “a collective term that embraces several clinical problems that involve the masticatory muscles, the temporomandibular joint (TMJ) and associated structures” in the Diagnostic Criteria for Temporomandibular Disorders (DC/TMD) [1]. TMD can be either (1) joint-related (arthralgia) or (2) muscle-related (myalgia) [1]. TMD signs include pain at palpation, popping, clicking, restricted opening, mandibular deviation during opening and closing, muscle soreness, headaches, and earaches [2]. According to a literature review [3], the prevalence of TMD varies between 10.6% and 68.1% in men and between 21.2% and 72.4% in women. A systematic review including 11 studies found a frequency between 3.4% and 65.7% for pain-related TMD signs and symptoms [4].

Excessive overloading of the TMJ beyond physiologic tolerance is one of the primary determinants for TMD [5], being often associated with TMJ degenerative disease and subsequent inflammation [6,7]. The osseous alterations include flattening, erosion, osteosclerosis, subchondral cysts, and formation of osteophytes [8,9]. Based on radiographic findings, the estimated prevalence of degenerative disease ranges from 8% to 35% [10]. TMJ degenerative disease may lead to alterations in condylar form and structure, resulting in mandibular asymmetries or deviations, canting of the occlusal plane or mandibular retrusion [11,12]. Dental and facial deformities, malocclusion, and limited mandibular movements may occur [13]. Teenagers and young adults may be particularly vulnerable to the detrimental consequences of degenerative disease, since degenerative processes can begin at a very young age [14]. Therefore, it is imperative to intercept and, if needed, treat degenerative processes promptly, as it was suggested also in another study [15].

TMJ is assessed using a variety of imaging methods since it is a unique joint with a biomechanically complex articular system [16]. Cone beam computed tomography (CBCT) is the preferred TMJ imaging method for osteoarthritis and other condylar degenerative disease, as well as dentoalveolar and skeletal parameters, due to its high reliability and precision for both volumetric and linear osseous measurements [17,18]. Another advantage of CBCT is represented by its unique characteristics of generating cephalometric two-dimensional (2D) images without exposing the patient to additional radiation [19]. Furthermore, compared to traditional multi-slice spiral computed tomography (CT), CBCT offers a high-resolution imaging of TMJ osseous structures while significantly reducing radiation exposure and costs [20]. 

Multiple treatment strategies for TMD have been described, from the conservative ones, such as physical therapy and occlusal splint [21], to the more advanced ones, such as botulinum toxin or complex TMJ surgery [22]. Among them, cranio-mandibular manual therapy is a noninvasive treatment option for TMD patients. It can be used alone or with occlusal splint [23]. Systematic reviews [24,25] emphasized the benefits of manual therapy in improving mandibular function and symptom relief in pain-related TMD. A systematic review [26] assessed the benefits of manual therapy compared to occlusal splint therapy in pain-related TMD, showing that both therapies were effective in symptom relief (*p* = 0.08; weighted standardized mean difference −0.29; 95% confidence interval [CI], −0.62 to 0.04). Rocabado’s 6 × 6 exercises were shown to be effective in enhancing masseter muscle elasticity, respectively, in reducing pain scores for TMD patients [27]. 

Occlusal splints are described as muscle deprogrammers or jaw repositioners [28], which promote condyle-fossa realignment, maxillo-mandibular ideal relationship, reduction of neuromuscular activity, and joint stabilization [29]. These action mechanisms unload the TMJ and allow the condyles to rest in a musculoskeletally stable position [28,30]. According to Ok et al. [31], occlusal splints induce a functional intra-articular loading on the anterior condylar head and anterior aspect of the glenoid fossa, promoting bone remodeling observed on CBCT images. Moreover, bone formation as “double contour” aspect in the medial and intermediate regions of the condyle was observed after occlusal splint therapy in 36 TMD patients [32]. A randomised controlled study [14] found a significantly higher rate of condylar repair in an occlusal splint group (78.1%/, 25/32 TMJ) when compared to a control group (48.6%/, 17/35 TMJ). Moreover, TMJ condylar regeneration with new bone formation was exclusively observed in an occlusal splint group (50%/, 16/32 TMJ), suggesting the benefits of using occlusal splints for biomechanical unloading.

The anterior repositioning splints [33] and stabilization splints [34] are commonly used in TMD management. Stabilization splints are manufactured in centric relation (CR) position and offer multiple and uniform static and dynamic teeth contacts. The CR is defined as “the maxillomandibular relationship in which the condyles articulate in the anterior-superior position against the posterior slopes of the articular eminences” [35], being the most stable and comfortable position for the jaws, in which the joints can be loaded without discomfort [36].

The minimum duration of occlusal splint therapy is three months, depending on the TMD initial pathology. Longitudinal studies investigating osseous changes associated with TMJ degenerative disease reported follow-up periods ranging from six months to one year [31,37,38]. Some of the studies have identified significant occlusal and skeletal changes following occlusal splint therapy [39,40]. On the contrary, other studies [41] have revealed minimal occlusal changes with no skeletal effects. These changes may benefit some skeletal profiles but could present challenges for others. It may be important to evaluate skeletal parameters alongside current diagnostic criteria before starting occlusal splint therapy in TMD patients, a hypothesis that requires further investigation. While occlusal splint therapy has shown significant clinical improvements in TMD patients, there is still insufficient data on radiographic changes, particularly regarding condylar remodeling and skeletal changes. Therefore, this study seeks to explore these aspects further, providing a comprehensive understanding of the therapeutic outcomes using occlusal splint therapy in TMD patients. 

This retrospective study aimed to assess the effects of occlusal splints in conjunction with cranio-mandibular manual therapy on osseous condylar changes and occlusal and skeletal changes in TMD patients. We hypothesized that combined therapy produces improvements in condylar remodeling, as well as skeletal and occlusal changes.

## 2. Methods

### 2.1. Study Design and Inclusion Criteria

Clinical data and CBCT imaging of patients attending the clinical department of “Iuliu Hatieganu” University of Medicine and Pharmacy for TMD between 2015 and 2017 were used for this retrospective cohort study. The data was collected from hard copies securely stored in the clinical department’s database, in compliance with data protection standards. Similarly, digital data stored on encrypted servers, accessible only to authorized personnel, was collected. At the moment of presenting in the clinical department for treatment, the patients provided informed consent for treatment and the use of their clinical and paraclinical data in further studies. The study was conducted following the Declaration of Helsinki, and the protocol was approved by the Ethics Committee of “Iuliu Hatieganu” University of Medicine and Pharmacy (no. 403/2 July 2015).

The main inclusion criteria in this study were patients with pain in jaw, in ear, or in front of ear who addressed for treatment, diagnosed with TMD based on the clinical data records following the Research Diagnostic Criteria for Temporomandibular Disorders (RDC/TMD) [1] checklist (pain-related signs as arthralgia or myalgia, intra-articular signs as clicking or crepitus) and treated with occlusal splint and cranio-mandibular manual therapy. The inclusion and exclusion criteria are listed in Table 1.

**Figure 1:**
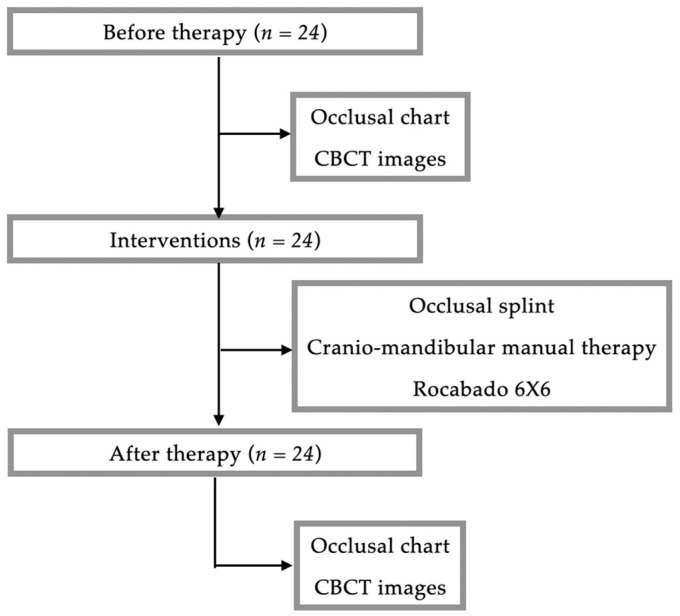
Flowchart of the participants included in this study.

Therefore, 24 participants—22 women and 2 men—were included with an average age of 23.88 ± 4.66 years. CBCT scans were performed prior to therapy and immediately at the end of the therapy as part of the clinical department standard protocol for patients with RDC/TMD positive diagnosis and managed with occlusal splint and cranio-mandibular manual therapy. CBCT images were used in this study. Similarly, static occlusion assessments were performed prior to therapy and immediately at the end of the therapy. Occlusal charts containing static occlusion parameters were used in this study (Figure 1).

### 2.2. Clinical and Paraclinical Interventions

A comprehensive clinical examination was conducted by an experienced orthodontist (I.B.) for each patient who sought treatment, starting with anamnesis of a positive history of pain in masticatory muscles or TMJ. According to RDC/TMD [1], clinical examination consisted of palpation of masticatory muscles (temporalis and masseter muscles) and TMJ (lateral and posterior pole) to evaluate muscle and joint pain. Clicking or popping and crepitus were also assessed.

Following RDC/TMD positive diagnosis, a methodological assessment for static occlusion parameters in right canine, left canine, and incisors overbite [42] was conducted by the same experienced orthodontist. Moreover, a CBCT scan of the cranium was carried out in a specialised imaging center. Every patient underwent occlusal splint therapy and cranio-mandibular manual therapy, which targeted pain-related signs and symptoms of masticatory muscles and TMJ. 

To manufacture occlusal splints, impressions of both arches (Cavex Cream Alginate, iDent, Chisinau, Republic of Moldova,) were performed together with an assisted bite registration for the CR position using a leaf gauge (Leaf Gauge, Huffman Dental, Springfield, Ohio, USA) and a facebow registration (Artex Facebow, Amann Girrbach AG, Pforzheim, Germany). All procedures were conducted by the same experienced orthodontist (I.B.). For determining the RC position, the leaf gauge was placed between the anterior incisors, and the patient was instructed to bite down on the leaf and to move the mandible forward (activating the lateral pterygoid muscles) and backward (activating the retrusion muscles and releasing the lateral pterygoids). Eventually, the patient was asked to lightly squeeze by activating the elevator muscles. The contraction of the elevator muscles associated with the relaxation of the protrusion muscles guided the condyles to the CR position [43]. A polyvinyl siloxane material (Occlufast Rock, Zhermack, Badia Polesine, Italy) was used to register this position.

Occlusal splints with a flat, occluding surface were manufactured using the semi-adjustable articulator (Artex CR, Amann 100 Girrbach AG, Pforzheim, Germany) in the patient’s arc of closure. Maxillary occlusal splints made of heat-cured acrylic material (Acrylic Vertex Orthoplast, Vertex Dental, Zetterberg, The Netherlands) were designed in a maxillo-mandible CR position with equal contacts in mandibular buccal cusps and incisal edges. Additionally, anterior guidance with immediate posterior disocclusion was designed [44]. The occlusal splints were divided into three sections: one anterior section from canine to canine and two posterior sections from premolars to molars. Every patient was instructed to wear the occlusal splint 24 h a day [45], except for brushing and eating: anterior segment during the nighttime; posterior segments during daytime, including during mastication; and the three segments together for three hours in the evening.

A physiotherapist with expertise in manual therapy performed cranio-mandibular therapy for the TMJ and masticatory muscles, which included the following techniques: (1) anterior glide and distraction of TMJ (gentle anterior gliding forces combined with joint pulling), (2) contract-relax stretching targeting the upper trapezius, levator scapulae and occipital, passive movement technique (mobilization of cervical spine), (3) neuromuscular re-education of deep neck flexors, (4) gentle stretching of bilateral masseter and temporalis, (5) intra-oral release of temporalis (gentle pressure to the tendonous insertions of the temporalis muscle while guiding the patient through incremental mouth opening to their maximum range), and (6) intra-oral release of medial and lateral pterygoid (gentle pressure into the pharyngeal mucosal tissues overlying the pterygoid origins) [46,47]. Manual therapy was recommended to reduce pain and muscle spasms, to realign the soft tissues, to enhance the drainage of synovial fluid, to reduce local ischemia, and to improve TMJ function. 

Additionally, all patients were given guidance and instructions on how to perform Rocabado’s 6 × 6 exercises. The manual therapist monitored the patient until the exercises were completed correctly. Afterwards, the patient was advised to perform the exercises daily at home. The Rocabado’s 6 × 6 exercises consisted of the following: (1) position of the tongue at rest, (2) shoulder girdle retraction, (3) upper cervical flexion stabilization, (4) lower cervical retraction, (5) controlled TMJ rotation, and (6) mandibular rhythmic stabilization [48]. 

Every two weeks, a manual therapy session and an occlusal splint checkup were performed over a three-month period. After this period, palpation of the masticatory muscles and TMJ was conducted. If pain-related signs and symptoms persisted, the combined therapy continued with additional manual therapy sessions, occlusal splint checkups, and palpation of the masticatory muscles and TMJ every two weeks. The combined therapy was considered complete after two successive pain-free palpations of muscles and TMJ and the resolution of the patient’s chief complaint. Immediately following therapy, the same experienced orthodontist (I.B.) performed the same methodological assessment for static occlusion parameters. Moreover, a CBCT scan of the cranium was carried out immediately at the end of therapy in the same specialised imaging center.

A CBCT device (CRANEX 3D, SOREDEX, Tuusula, Finland) was used for the primary acquisition. The parameters of exposition were 80 kilovoltage (kV), 6.0–8.0 milliamperage (mA), 400 µm voxel size, exposition time of 15 s, and 23 cm × 17 cm field of view (FOV). The patient was seated, the head was straight with the Frankfort plane parallel to the floor, and the teeth were closed to maximal intercuspation. The As Low As Reasonably Achievable (ALARA) principle was adhered to in all CBCT examinations.

TMD signs and symptoms, static occlusion parameters, the number of occlusal splint checkups, and manual therapy sessions, as well as treatment duration, were recorded in the patient’s file.

**Figure 2:**
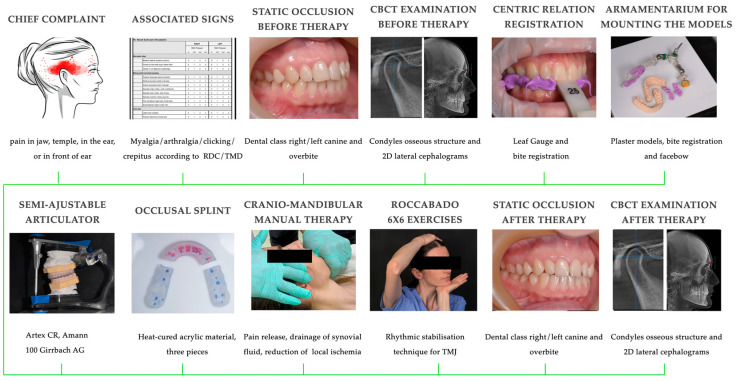
The protocol of the clinical and paraclinical interventions. RDC/TMD—Research Diagnostic Criteria for Temporomandibular Disorders, CBTC—cone beam computed tomography, TMJ—temporomandibular joint.

The protocol of the clinical and paraclinical interventions is illustrated in Figure 2.

### 2.3. Outcomes Assessed

Firstly, condylar remodeling changes after therapy were evaluated using CBCT images. Secondly, vertical and sagittal skeletal and occlusal changes after therapy were assessed using lateral cephalograms generated from CBCT images and occlusal charts.

#### 2.3.1. CBCT Analysis

In order to analyse the CBCT data, both soft- and hard-tissue tridimensional (3D) renderings from CBCT scans were oriented in the visual axis-simulated natural head position, which is based on a true horizontal line depicting the subject looking at a distant point at eye level. The 3D orientation used Frankfurt horizontal, midsagittal, and transporionic planes. The Frankfurt plane was defined by the porion and orbitale landmarks, the midsagittal plane by nasion, anterior nasal spine, basion, and the transporionic plane by porion landmarks perpendicular to the Frankfurt plane. The volume was rotated in sagittal, axial, and coronal views to align these planes correctly [49].

##### Osseous Condylar Structure

**Figure 3:**
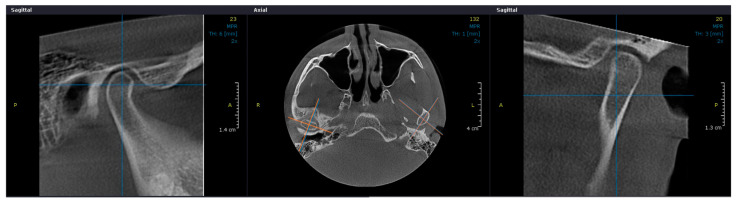
CBCT images selection for morphological assessment: the axial slice with the largest mediolateral diameter of the condylar head with a sagittal slice crossing in the middle of the distance between the most prominent points on medial and lateral poles and perpendicular to the coronal axis.

On reconstructed CBCT, multiple sagittal images at 0.3 mm slice intervals were used to assess the condyle’s osseous features. OnDemand3DViewer version 1.0 (Cybermed Inc., Seoul, Republic of Korea) was used to analyse the images. Two specialists in oral medicine (A.B. and M.T.), who were blinded to the clinical diagnoses as well as treatment assignments and outcomes, selected the axial slice with the largest mediolateral diameter of the condylar head to cut next to a sagittal slice crossing in the middle of the distance between the most prominent points on medial and lateral mandibular condylar poles and perpendicular to the coronal axis (Figure 3).

Applying a filter on 0.3 mm sagittal oblique reformatted images, the examiners assessed the following TMJ osseous structures: (1) normal, or no morphological change with convex condylar shape, uninterrupted cortical bone, or no cavity in bone marrow; (2) flattening of the condyle’s anterior region, or loss of its convex form; (3) erosion, or the articular cortical bone’s loss of continuity; and (4) subchondral cysts, or the creation of cavities beneath the articular surface that differ from the typical pattern of bone marrow (Table 2).

In order to improve the evaluation of osseous structures, at least two out of three consecutive mid-sagittal slices had to present the relevant osseous structure. The assessment was done for both TMJ, for all the patients. The two examiners evaluated and compared the CBCT images in a single observation session. In case of disagreement between the evaluators, they reexamined together the images to guarantee accurate assessment. The images were collected from CBCT scans performed prior to therapy and at the end of therapy. 

Even if the loss of the condyle’s anterior region convex shape (or flattening) is not classified as a degenerative joint disease, according to the International Research Diagnostic Criteria for Temporomandibular Disorders Consortium Network and the Orofacial Pain Special Interest Group [1], we included flattening as an osseous morphological structure of the TMJ, but it was not taken into consideration in the statistical analysis when comparing the morphological changes after therapy.

##### Cephalometric Parameters

**Figure 4:**
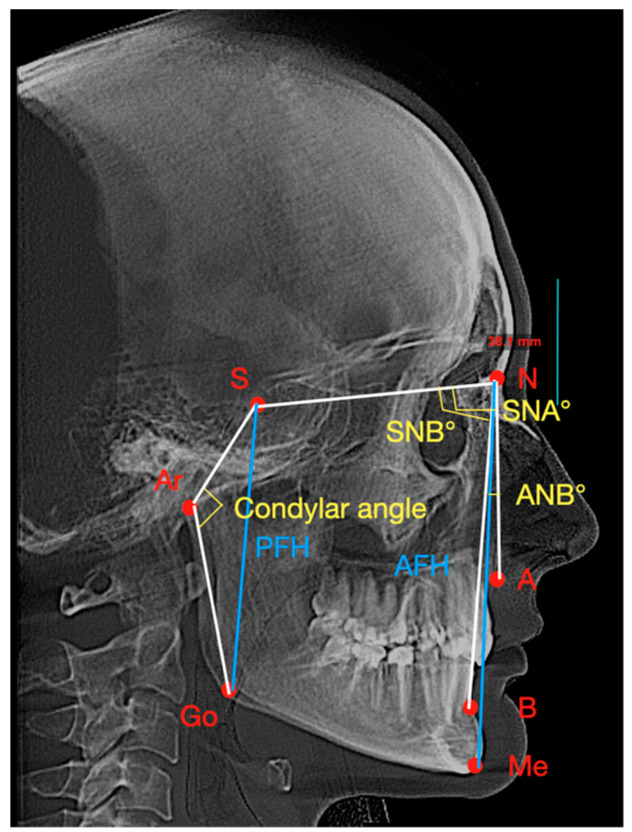
List of cephalometric points and parameters: Subspinale—Point A, Supramentale—Point B, Sella—S, Nasion—N, Menton—Me, Gonion—Go, Articulare—Ar, Sella-Nasion-A point—SNA, Sella-Nasion-B point—SNB, A point-Nasion-B point—ANB, Sella-Articulare-Gonion—condylar angle, anterior facial height—AFH, posterior facial height—PFH.

By using OnDemand3DViewer version 1.0 (Cybermed Inc., Seoul, Republic of Korea) for lateral radiographic projection of the entire volume, one right 2D lateral cephalogram was generated for each CBCT scan. A dedicated software (Dolphin Imaging Cephalometric and Tracing Software, version 11.9, Chatsworth, CA, USA) was then used to trace each lateral cephalogram. The same two specialists in oral medicine (A.B. and M.T.) selected the following cephalometric points: Subspinale (point A), Supramentale (point B), Sella (S), Nasion (N), Menton (Me), Gonion (Go), and Articulare (Ar). After, the following parameters were measured according to Steiner method [50]: Sella-Nasion-A point (SNA), Sella-Nasion-B point (SNB), A point-Nasion-B point (ANB), Sella-Articulare-Gonion (condylar angle), anterior facial height (AFH), and posterior facial height (PFH). The cephalometric parameters were used to determine if there are changes in the vertical and sagittal positions of the mandible after occlusal splints in conjunction with cranio-mandibular manual therapy in TMD patients. Table 3 and Figure 4 contain the list of cephalometric points and parameters.

Each measurement was performed twice, with a one-week washout interval, and the images were analyzed randomly without access to prior measurements. The intra-observer and inter-observer correlation coefficients (ICC) were assessed with 95% confidence intervals for each measurement.

#### 2.3.2. Static Occlusion Parameters

Furthermore, the occlusion parameters collected from the occlusal charts were used to assess the position of the mandible following therapy (Table 4).

### 2.4. Statistical Analysis

Version 4.1.2 of the R environment for statistical computation and graphics (R Foundation for Statistical Computation, Vienna, Austria) was used for the statistical analysis [51]. Normal distribution of data was checked visually via quantile–quantile (QQ) plots and using Shapiro–Wilk test. The mean and standard deviations (for regularly distributed data) and the medians and Q1–Q3 interquartile ranges (for non-normally distributed data) were computed, respectively. The intra- and inter-observer reliability for cephalometric parameters was assessed using ICC with 95% confidence intervals. Stuart–Maxwell (for categorical nominal data), McNemar test (for paired nominal data), Wilcoxon’s signed rank test (for non-normally distributed data), and the paired *t*-test (for regularly distributed data) were used to evaluate the statistical differences between parameters before and after therapy. Two-tailed *p* < 0.05 was chosen as the statistical significance threshold. The effect size for the paired *t*-test was computed using Cohen D [52]. Wilcoxon effect size was computed using R according to Rosenthal et al. [52]. Cohen G was used for McNemar test effect size estimation [53]. The Cohen D and R are classified as trivial effect (values less than 0.2), small effect (values between 0.2 and 0.5), medium effect (values between 0.5 and 0.8), and large effect (values higher than 0.8) [53]. The Cohen G is classified as very small effect (values less than 0.05), small effect (values between 0.05 and 0.15), medium effect (values between 0.15 and 0.25), and large effect (values higher than 0.25) [52,53].

## 3. Results

The 24 participants included in this study presented the following signs according to RDC/TMD, along with pain in jaw, in ear, or in front of ear (Table 5).

Occlusal splint checkups and manual therapy sessions ranged from 6 to 26 times, with an average of 14.83 times *±* 6.53. The occlusal splint was worn for an average time of 7.42 ± 3.27 months (range from 3 to 13 months) until the subjective chief complaint (pain in jaw, temple, in ear, or in front of ear) and the associated pain-related signs (myalgia and arthralgia) disappeared during two successive clinical evaluations.

### 3.1. Condylar Remodeling

The normal osseous structure was present in 3 out of 24 right TMJ (12.5%) and 4 out of 24 left TMJ (16.6%). The number of TMJ presenting normal osseous structures did not change following the therapy. The loss of condyle’s anterior region convexity was present in 21 out of 24 right TMJ (87.5%) and 20 out of 24 left TMJ (83.3%). The number of TMJ with condylar flattening did not change following therapy.

Before the onset of treatment, condylar erosion was present in 10 out of 24 right TMJ (41.6%) and in 6 out of 24 left TMJ (25%). Following therapy, 6 right TMJ (60%) and 4 left TMJ (66.6%) had a complete remodeling. The number of TMJ affected by condylar erosion significantly decreased after treatment (*p* = 0.01 and *p* = 0.05, McNemar Test).

Before the onset of therapy, subchondral cysts were present in 9 out of 24 right TMJ (37.5%) and in 8 out of 24 (33.3%) left TMJ, respectively. At the end of treatment, 6 right TMJ (66,6%) and 5 left TMJ (62.5%) had a complete remodeling. The number of TMJ affected by subchondral cysts significantly decreased after treatment (*p* = 0.02, *p* = 0.05, McNemar Test). A large effect size for the improvement in surface erosion and subchondral cyst was observed after therapy (Cohen G > 0.25) (Table 6).

Bilateral condylar flattening without surface erosion or subchondral cysts was present in two subjects (8.33%) before and after therapy. Before the onset of treatment, unilateral surface erosion or subchondral cyst was present in 11 subjects (45.83%). At the end of treatment, 6 subjects had complete healing (54.54%), and 5 subjects (45.45%) still had unilateral surface erosion or subchondral cysts. Before the onset of treatment, bilateral surface erosion or subchondral cysts was present in 11 subjects (45.83%). At the end of treatment, 6 subjects (54.54%) had complete healing (54.54%), 4 subjects (36.36%) had unilateral surface erosion or subchondral cysts, and 1 subject still had bilateral surface erosion or subchondral cysts (9.09%). Both in bilateral and unilateral pathology was observed a complete remodeling and healing in a statistically significant manner (*p* = 0.001, Stuart-Maxwell test) (Table 7).

**Figure 5:**
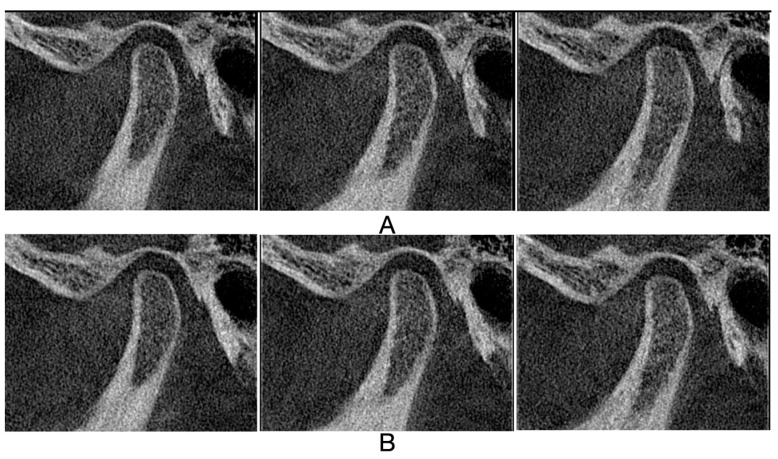
CBCT oblique sagittal view: (**A**) CBCT images before therapy: first, second, and third mid-sagittal slice of left condyle, no morphological changes: convex condylar shape, uninterrupted cortical bone, no cavity in the bone marrow; (**B**) CBCT images after therapy: first, second, and third mid-sagittal slice of left condyle, no morphological changes.

**Figure 6:**
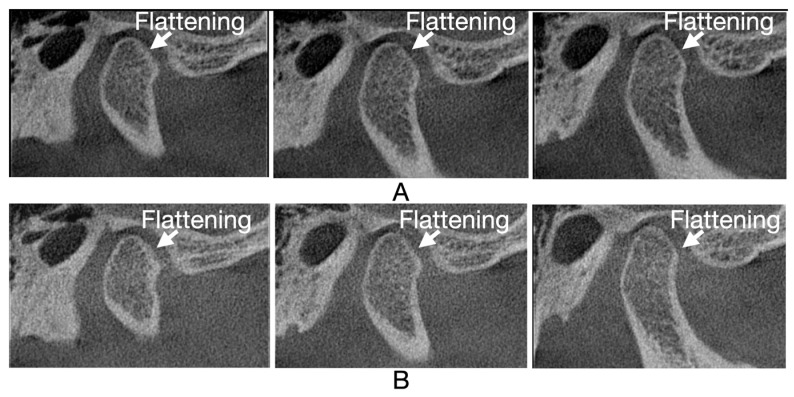
CBCT oblique sagittal view: (**A**) CBCT images before therapy: first, second, and third mid-sagittal slice of right condyle, loss of condyle’s anterior region convex shape; (**B**) CBCT images after therapy: first, second, and third mid-sagittal slice of right condyle, flattening.

**Figure 7:**
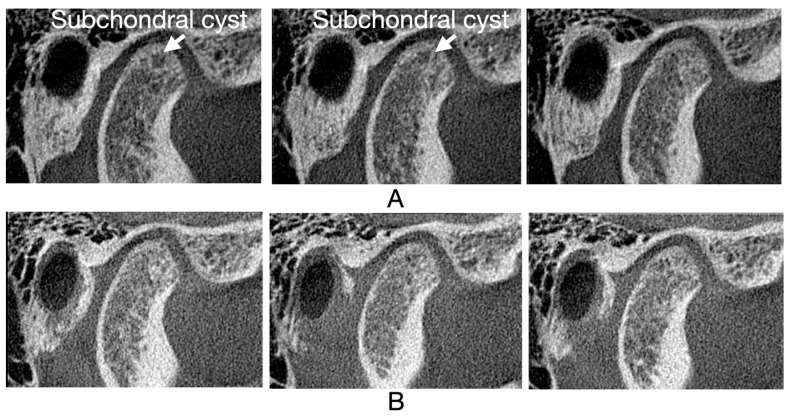
CBCT oblique sagittal view: (**A**) CBCT images before therapy: first, second, and third mid-sagittal slice of right condyle, typical pattern of cavities in bone marrow; (**B**) CBCT images after therapy: first, second, and third mid-sagittal slice of right condyle, healed with no cavities in the bone marrow.

**Figure 8:**
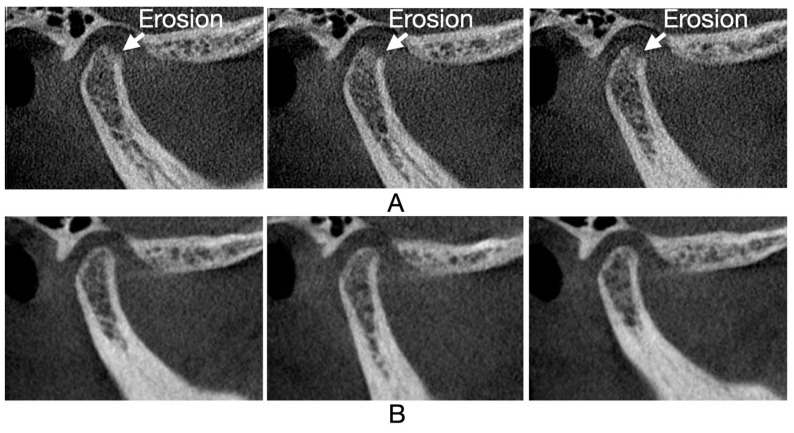
CBCT oblique sagittal view: (**A**) CBCT images before therapy: first, second, and third mid-sagittal slice of right condyle, loss of continuity in the cortical bone; (**B**) CBCT images after therapy: first, second, and third mid-sagittal slice of right condyle, healed with continuity in the cortical bone.

Figure 5, Figure 6, Figure 7 and Figure 8 illustrate three consecutive CBCT images with the following osseous structures of the condyle: normal, flattening, condylar erosion, and subchondral cyst before and after therapy.

### 3.2. Vertical and Sagittal Skeletal Changes

For cephalometric parameters measured on the CBCT images, the ICC with 95% confidence intervals for intra-observer reliability for the first examiner (A.B.) were: 0.951 (95% CI 0.869–0.986, *p* < 0.001) for SNA, 0.874 (95% CI 0.836–0.91, *p* < 0.001) for SNB, 0.926 (95% CI 0.842–0.944, *p* < 0.001) for ANB, 0.901 (95% CI 0.814–0.946, *p* < 0.001) for condylar angle, 0.935 (95% CI 0.857–0.968, *p* < 0.001) for AFH, and 0.862 (95% CI 0.805–0.893, *p* < 0.001) for PFH. The ICC with 95% confidence intervals for intra-observer reliability for the second examiner (M.T.) were: 0.910 (95% CI 0.810–0.949, *p* < 0.001) for SNA, 0.918 (95% CI 0.853–0.941), *p* < 0.001) for SNB, 0.822 (95% CI 0.767–0.852, *p* < 0.001) for ANB, 0.872 (95% CI 0.806–0.916, *p* < 0.001) for condylar angle, 0.905 (95% CI 0.865–0.924, *p* < 0.001) for AFH, and 0.833 (95% CI 0.801–0.924, *p* < 0.001) for PFH.

The ICC with 95% confidence intervals for inter-observer reliability were: 0.814 (95% CI 0.788–0.89, *p* < 0.001) for SNA, 0.912 (95% CI 0.75–0.916, *p* < 0.001) for SNB, 0.912 (95% CI 0.83–0.906, *p* < 0.001) for ANB, 0.915 (95% CI 0.803–0.906, *p* < 0.001) for condylar angle, 0.863 (95% CI 0.818–0.916, *p* < 0.001) for AFH, and 0.806 (95% CI 0.722–0.83, *p* < 0.001) for PFH.

Following therapy, the sagittal position of the mandible significantly changed by a more distal position: SNB significantly decreased from 75.61 ±3.47° to 74.82 ± 3.41° (*p* = 0.02, paired *t*-test and Cohen D = 0.73, medium effect size), and ANB significantly increased from 4.05° (3.35–4.9°) to 4.8° (3.3–6.12°) (*p* < 0.001, Wilcoxon’s signed rank test and R = 0.48, small effect size). The vertical position of the mandible significantly changed by an increase in facial height: AFH significantly increased from 112.85 mm (109.28–118.72 mm) to 115.3 mm (112.58–118.88 mm) (*p* < 0.001, Wilcoxon’s signed rank test, R = 0.58, medium effect size), PFH/AFH ratio significantly decreased from 64.17 (61.39–66.1) to 63 (59.68–64.51) (*p* = 0.012, Wilcoxon’s signed rank test, R = 0.36, small effect size), and condylar angle significantly increased from 140.84 ± 8.18° to 144.42 ± 8.87° (*p* = 0.007, paired *t*-test, Cohen D = 0.6, medium effect size). Table 8 contains the means and medians of cephalometric parameters before and after treatment and the results of statistical tests, respectively.

Based on the initial condition of the degenerative disease (unilateral or bilateral pathology), we performed a subgroup analysis to assess the changes in the sagittal and vertical position of the mandible. Both in unilateral and bilateral pathology groups, the sagittal and vertical position of the mandible significantly changed by a more distal position and by an increase in facial height, respectively (*p* < 0.05). Table 9 contains the means and medians of cephalometric parameters before and after treatment for both groups and the results of statistical tests, respectively.

### 3.3. Vertical and Sagittal Occlusion Changes

Before the onset of treatment, on the right side, canine dental class I was present in 11 subjects (45.83%), class II with one-half cusp distalization was present in 11 subjects (45.83%), class II with one cusp distalization was present in one subject (4.17%), and class III with one-half cusp mesialization was present in one subject (4.17%). At the end of treatment, 4 subjects (36.36%) remained in dental class I, 6 subjects (54.55%) changed to class II with one-half cusp distalization, and 1 subject (9.09%) changed to class III with one-half cusp mesialization. Eight subjects (72.73%) remained in class II with one-half cusp distalization, and 3 subjects (27.27%) changed to class II with one cusp distalization. One subject changed from class II with one cusp distalization to class II with one-half cusp distalization (100%). One subject changed from class III with one-half cusp mesialization to class I (100%). The static occlusion for right canines significantly changed by a more distal relationship after treatment (*p* = 0.038, Stuart–Maxwell test).

Before the onset of treatment, on the left side, canine dental class I was present in 12 subjects (50%), class II with one-half cusp distalization was present in 9 subjects (37.5%), and class II with one cusp distalization was present in 3 subjects (12.5%). At the end of treatment, 3 subjects (25%) remained in class I, and 9 subjects (75%) changed to class II with one-half cusp distalization. Seven subjects (77.78%) remained in class II with one-half cusp distalization, 1 subject (11.11%) changed to class II with 1 cusp distalization, and 1 subject (11.11%) changed to class III with one-half cusp mesialization. Out of 3 subjects presenting class II with one cusp distalization, 1 subject (33.33%) changed to class I, and 2 subjects (66.66%) changed to class III with one-half cusp mesialization. The static occlusion for left canines significantly changed by a more distal relationship after treatment *(p* < 0.001, Stuart–Maxwell test).

Before the onset of treatment, 17 subjects (70.83%) presented normal overbite, 5 subjects (20.83%) presented deep bite, and 2 subjects (8.33%) presented open bite. At the end of treatment, 15 out of 17 subjects (88.24%) presenting normal overbite changed to open bite, 3 out of 5 subjects (60%) presenting deep bite changed to normal overbite, and 2 out of 2 subjects (100%) remained deep bite. The static occlusion significantly changed by a decrease in the anterior overbite after treatment (*p* < 0.001, Stuart–Maxwell test). Table 10 presents the static occlusion parameters for the right canine, left canine, and incisors before and after therapy.

## 4. Discussion

The first null hypothesis was accepted since 21 out of 33 degenerative TMJ (63.6%) presented complete remodeling and healing in a statistically significant manner: 10 out of 16 TMJ with condylar erosion (62.5%, *p* < 0.02) and 11 out of 17 TMJ with subchondral cysts (64.7%, *p* < 0.05). Condylar remodeling had a large effect size according to Cohen G value. Similarly, in both unilateral and bilateral pathologies, it was observed a significant complete condylar remodeling in 6 out of 11 subjects presenting unilateral surface erosion or subchondral cysts (54.54%) and 6 out of 11 subjects presenting bilateral pathology (54.54%). Similar results were found in a study conducted on 89 TMD patients with condylar degenerative processes treated with occlusal splint therapy, showing a complete remission in 94 out of 179 TMJ (52.5%) over a period of 21 ± 10 months [13]. Another prospective study showed improvements in condylar erosion, sclerosis, and subchondral cysts ranging from 57.5–100% using a full-coverage splint over a period of 10.5 ± 2.4 months [37]. While these studies observed condylar remodeling over extended periods of time, others reported shorter time therapy of 6 months [32,38,41]. After splint therapy, adaptive bone remodeling as a “double contour” aspect occurred in 80% of patients (29 out of 36) over a period of 5.5 ± 2.7 months [32]. Similarly, in young individuals with early stages of degenerative processes, Lei et al. [41] reported a condylar remodeling of 62.7% (42 out of 67 TMJ) over a period of 6 months: a remodeling of 78.1% using the anterior repositioning splint and a remodeling of 48.6% without any therapy. The average time for condylar remodeling was 7.42 ± 3.27 months in our study. The variability in follow-up periods for the resolution of TMJ degenerative disease may be attributed to age-related differences in bone repair capacity [54], as well as the inability to precisely determine the onset of degenerative disease.

The mechanical loading on the condylar head and glenoid fossa is one major cause of TMJ degenerative disease [55]. Functional loading promotes cartilage integrity and regeneration, while excessive pressure can disturb joint lubrication and increase intra-articular pressure [37]. In contrast, Matsumoto et al. [56] concluded that biomechanical stress or inflammation on the glenoid fossa promoted bone thickness. Occlusal splints induce anterior and downward condylar displacement, changing joint space stress and mechanical loading on osseous structures [32,57]. This mechanism stimulates bone formation on specific condylar surfaces in TMD patients, according to Kim et al. [57]. Similarly, Ok et al. [37] concluded that splint therapy allowed for adaptive bone changes by extending the distance in particular load-bearing sites.

Our study suggests that therapeutic strategies in TMD associated with degenerative disease should promote bone remodeling in addition to pain-related signs and symptoms and function recovery. In this context, low-dose irradiation techniques (CBCT) could be used to assess the therapeutic effects of degenerative disease.

Erosive condylar surface and subchondral cyst can, over an extended period of time, heal and regain uninterrupted cortical bone with no cavity in bone marrow [37]. This is commonly associated with condylar remodeling characteristics, such as the flattening of condylar convexity, but with the capacity to withstand loading during jaw function [58]. In this study, before therapy, 41 out of 48 TMJ (85.4%) showed condylar flattening. The number was preserved following the therapy. The cortical lining and the marrow bone quality may be more important in assessing the health of the TMJ and the disease’s condition rather than morphological abnormalities caused by bone damage throughout the healing process.

The second null hypothesis was accepted since significant changes in the vertical and sagittal position of the mandible were observed following occlusal splint therapy in conjunction with cranio-mandibular manual therapy in TMD patients.

After therapy, the sagittal position of the mandible changed by a significant retrusion of the mandible (significant decrease of SNB angle, *p* = 0.02) and a significant increase in the sagittal discrepancy between maxilla and mandible (significant increase of ANB angle, *p* < 0.001). Similarly, significant occlusion changes toward a more distal relationship were observed in the right and left canines after treatment (*p* = 0.038 and *p* = 0.038). After therapy, the vertical position of the mandible changed by a significant increase in facial height: significant increase of AFH and condylar angle (*p <* 0.001 and *p =* 0.007), and significant decrease of PFH/AFH ratio (*p* = 0.012). Similarly, significant occlusion changes toward a decrease in the anterior overbite were observed (*p* < 0.001).

Similar results were found on 40 TMD subjects who underwent occlusal splint therapy and manual therapy: the vertical position of the mandible significantly increased (*p* < 0.0001), while its sagittal position significantly decreased (*p* = 0.0065) [59]. Another retrospective study on 74 TMD patients with myofascial and/or intra-articular pathology treated with occlusal splint therapy resulted in skeletal and dentoalveolar changes: a significant decrease in the incisors’ overbite (difference: −0.54 ± 0.83 mm), a significant decrease in mandibular position according to SNB (difference: 1.60 ± 1.36°), and a decrease in facial height according to PFH/AFH ratio (difference: 0.45% ± 1.07) [39]. On the contrary, a prospective study involving 69 TMD patients managed with an anterior repositioning splint a mild, temporary posterior open bite with no subsequent skeletal changes [41]. Similarly, a meta-analysis [60] concluded that fixed functional appliances did not cause any skeletal changes. Additionally, factors related to age, muscle function, and genetics may play an important role in skeletal and dentoalveolar changes [61].

Several parameters assessed by CBCT were predictors with high sensitivity and specificity for anterior open bite development after occlusal splint therapy in a prospective study including 87 TMD patients: the distance between the mesio-buccal cusp tip of the right mandibular first molar and the mandibular plane (cutoff value of 31.795 mm), interincisal angle (cutoff value of 123.44°), PHF/AFH ratio (cutoff value of 62.5%), ramus height (cutoff value of 43.365 mm), and Sella to Articulare (cutoff value of 21.69 mm) [62]. Moreover, a decrease of SNB angle or PFH/AFH ratio and an increase of ANB angle, AFH, or condylar angle may be advantageous for dental and skeletal class III, deep bites, and hypodivergent profiles, while they are problematic for dental and skeletal class I and II, minimum overbites or open bites, and hyperdivergent profiles. Therefore, skeletal and dentoalveolar parameters should be considered in addition to current RDC/TMD diagnostic criteria to provide a comprehensive understanding of the therapeutic outcomes before starting occlusal splint therapy in TMD patients.

The new orthopedically stable position of the mandible represents a key factor in determining the appropriate management of TMD following occlusal splint therapy. Occlusal equilibration treatments, such as orthodontics, prosthodontics, or selective grinding, can be required as follows: intrusion of the molars in the case of an anterior open bite [63] or selective grinding of the molars’ inclines in the case of condylar displacement from the stable CR position [64].

Our study showed that pain-related signs and symptoms entirely disappeared over an average period of 7.42 ± 3.27 months with a range from 3 to 13 months. An extensive range of treatment times with a different treatment period for each patient may be explained by each patient’s individual response and adaptability to therapy. Therefore, personalized treatment protocols to enhance patient outcomes in pain-related TMD should be considered. Derwich et al. [65] conducted a prospective case-control study involving 44 TMD patients and found that combined occlusal splint therapy and manual therapy were beneficial in alleviating pain and improving mandibular range of maximum motion. These findings are consistent with our findings. Furthermore, a meta-analysis of 52 randomized controlled trials [66] for painful TMD of muscular origin found effectiveness of 83.5% (low-quality evidence) for manual therapy and 71.7% (moderate-quality evidence) for occlusal splints. A meta-analysis [21] of 48 randomized controlled trials found that hard stabilization splints had an effectiveness of 59.7% (moderate-quality evidence) in pain reduction for myelogenous TMD. For arthrogenous TMD, the pain reduction was 52.9% (moderate-quality evidence) with hard stabilization splints alone.

This study has several limitations. Firstly, the sample size was small. Given the study’s retrospective nature, the daily usage time of the occlusal splint and the adherence to home exercises were reliant on patient self-reporting. The fact that Rocabado’s 6 × 6 home exercises were only related in the patient’s report without any daily recording data is another study limitation. A daily journal or a phone application should be taken into consideration for future potential research. However, pain-related signs and symptoms, the number of occlusal splint checkups, and the number of manual therapy sessions, as well as treatment duration, were well documented in the patient’s file. The treatment assignment investigator (I.B.) was not specifically calibrated for this study, which may introduce a potential bias in the clinical assessments. Although the two investigators involved in the assessment process (A.B. and M.T.) were blinded to the clinical diagnoses, as well as treatment assignments and outcomes, they were not specially calibrated for this study. Nevertheless, to ensure accurate assessment of condylar changes, three successive mid-sagittal slices were evaluated independently by each investigator, and in case of disagreement, the images were reexamined collaboratively. Furthermore, the intra- and inter-reliability of cephalometric parameters revealed good to excellent validity scores. As this study employed a retrospective scenario, it cannot be conclusively stated that the observed changes were solely attributable to the interventions. Furthermore, the lack of a control group (such as no treatment or splint therapy alone) or a standardized patient-reported outcome measure (such as pain scales or quality of life measures) makes it more challenging to ascertain whether the combined splint and manual therapy or each therapy individually was more effective in alleviating pain, particularly over prolonged use. Future randomized controlled trials should involve a larger sample size, include a control group, and have extended follow-up periods to evaluate both the treatment effectiveness over time and the potential recurrence of degenerative disease after completing occlusal splint therapy.

To our knowledge, this is the first study to evaluate the effects of occlusal splint and cranio-mandibular manual therapy on both condylar remodeling and occlusal and skeletal changes in TMD patients. The dual therapeutic benefit of promoting condylar remodeling, along with pain-related signs and symptom alleviation, supports the clinical utility of this combined therapy. Understanding long-term osseous changes in TMD patients through CBCT evaluation may enhance the prognosis of degenerative disease and eventually prevent its irreversible effects. Early diagnosis and intervention are crucial, especially in managing a complex pathology such as TMD in young patients.

Moreover, this study found significant changes in both the vertical and sagittal position of the mandible, highlighting the broader impact of the combined therapy on the entire oral cavity and its dental parameters. In addition to RDC/TMD diagnostic criteria, skeletal and dental occlusion evaluations are necessary before occlusal splint therapy.

Potential condylar remodeling, along with skeletal and occlusal changes, underscores the need for a comprehensive approach in TMD to enhance patient outcomes.

## 5. Conclusions

Condylar degenerative disease presented significant remodeling (no condylar erosion, no subchondral cyst) after combined therapy over a period of 7.42 ± 3.27 months. A comprehensive TMD approach should focus on both symptom relief and bone remodeling to prevent irreversible consequences of TMJ degenerative disease.

The position of the mandible presented significant retrusion and increase in facial height following combined therapy. Skeletal and dental occlusion evaluation should be considered in addition to the current TMD diagnostic criteria before occlusal splint therapy in TMD patients.

Further randomized controlled trials are required to validate these findings and to explore the long-term implications of combined therapy in TMD management.

## Figures and Tables

**Table 1:** Inclusion and Exclusion Criteria for This Retrospective Study.

Inclusion Criteria	Exclusion Criteria
Pain in jaw, temple, in ear, or in front of ear	History of head or neck trauma
Associated pain-related signs according to RDC/TMD guidelines (arthralgia and myalgia)	Previous orthodontic or orthognathic treatment
Associated intra-articular signs according to RDC/TMD guidelines (clicking/popping/crepitus)	Presence of systematic diseases (e.g., autoimmune diseases, rheumatoid arthritis)
Older than 18 years old	Younger than 18 years old
Three or more stable pairs of opposing teeth in central incisors, premolars, and molars on both left and right side	Less than three stable pairs of opposing teeth in central incisors, premolars, and molars on both left and right side

RDC/TMD—Research Diagnostic Criteria for Temporomandibular Disorders.

**Table 2:** Classification of Osseous Structures on CBCT Image.

Osseous Structure	Definition
Normal	No morphological changes: convex condylar shape, uninterrupted cortical bone, or no cavity in bone marrow
Flattening	Loss of condyle’s anterior region convex form
Erosion	Loss of continuity in the cortical bone
Subchondral cyst	Typical pattern of cavities in bone marrow

**Table 3:** List of Cephalometric Points and Parameters.

Position of the Mandible	Point/Angle	Definition
	Point A	Subspinale—point localized in the deepest area of the anterior outline of the maxilla, below the anterior nasal spine
	Point B	Supramentale—point localized in the deepest area of the anterior outline of the mandible, above the pogonion
	Point S	Sella—geometrical center of Sella turcica
	Point N	Nasion—the most anterior point localized in the frontonasal suture
	Me	Menton—lowest point on the mandibular symphysis
	Go	Gonion—most posterior inferior point on angle of mandible
	Ar	Articulare—junction between inferior surface of the cranial base and the posterior border of the ascending rami of the mandible
sagittal position of the mandible	SNA (°)	Sella–Nasion–A point
	SNB (°)	Sella–Nasion–B point
	ANB (°)	A point–Nasion–B point
vertical position of the mandible	AFH (mm)	N–Me
	PFH (mm)	S–Go
	PFH/AFH (%)	PFH/AFH x 100
	Condylar angle (°)	S–Ar–Go

**Table 4:** List of Occlusion Parameters.

Position of the Mandible	Canines/Incisors	Definition
sagittal	Class I (1)	the lower canine occluded between the upper canine and the lateral incisor
	Class II with one-half cusp distalization (D ½ c)	the upper canine occludes with the lower canine
	Class II with one cusp distalization (D 1 c)	the lower canine occludes between the upper canine and the upper first premolar
	Class III with one-half cusp mesialization (M ½ c)	the upper canine occludes with the first lower premolar
	Class III with one cusp mesialization (M 1 c)	the upper canine occludes with the first and second lower premolar
vertical	Normal overbite (NOB)	upper incisors vertically overlap 1/3–1/2 of the lower incisors
	Deep bite (DB)	upper incisors vertically overlap more than 1/3–1/2 of the lower incisors
	Open bite (OB)	upper incisors vertically overlap less than 1/3–1/2 of the lower incisors

**Table 5:** Distribution of TMD Signs Among Included Patients According to RDC/TMD Criteria.

Category	Signs According to RDC/TMD	(%)
Associated pain-related signs	Arthralgia and myalgia	14/24 (58.33%)
	Arthralgia	10/24 (41.66%)
Associated intra-articular signs	Clicking/popping during opening and closing and during protrusive/laterotrusive movements (among arthralgia and myalgia patients)	8/14 (57.1%)
	Crepitus during opening/protrusive/laterotrusive movements (among arthralgia and myalgia patients)	6/14 (42.9%)
	Clicking/popping during opening and closing and during protrusive/laterotrusive movements (among arthralgia patients)	6/10 (60%)
	Crepitus during opening/protrusive/laterotrusive movements (among arthralgia patients)	4/10 (40%)

RDC/TMD—Research Diagnostic Criteria for Temporomandibular Disorders.

**Table 6:** Osseous Structures Before and After Treatment.

	Before Therapy	After Therapy	*p*-Value	Cohen G-Value
Right TMJ				
Normal	3/24 (12.5%)	3/24 (12.5%)	NaN *	NaN *
Flattening	21/24 (87.5%)	21/24 (87.5%)	NaN *	NaN *
Surface erosion	10/24 (41.6%)	4/24 (16.6%)	0.01 **	1 ***
Subcortical cyst	9/24 (37.5%)	3/24 (12.5%)	0.02 **	1 ***
Left TMJ				
Normal	4/24 (16.6%)	4/24 (16.6%)	NaN *	NaN *
Flattening	20/24 (83.3%)	20/24 (83.3%)	NaN *	NaN *
Surface erosion	6/24 (25%)	2/24 (8.33%)	0.05 **	1 ***
Subcortical cyst	8/24 (33.3%)	3/24 (12.5%)	0.05 **	0.71 ***

TMJ—temporomandibular joints, * NaN—data is not applicable, ** McNemar test, *** effect size for McNemar test.

**Table 7:** Unilateral and Bilateral Osseous Structures Before and After Therapy.

Before Therapy	After Therapy
No surface erosion/subcortical cyst: 2/24 (8.33%) =>	No surface erosion/subcortical cyst: 2/2 (100%)
	Unilateral surface erosion/subcortical cyst: 0/2 (0%)
	Bilateral surface erosion/subcortical cyst: 0/2 (0%)
Unilateral surface erosion/subcortical cyst: 11/24 (45.83%) =>	No surface erosion/subcortical cyst: 5/11 (45.45%)
	Unilateral surface erosion/subcortical cyst: 6/11 (54.54%)
	Bilateral surface erosion/subcortical cyst: 0/24 (0%)
Bilateral surface erosion/subcortical cyst: 11/24 (45.83%) =>	No surface erosion/subcortical cyst: 6/11 (54.54%)
	Unilateral surface erosion/subcortical cyst: 4/11 (36.36%)
	Bilateral surface erosion/subcortical cyst: 1/11 (9.09%)
*p* = 0.001 ^a^	

^a^ Stuart-Maxwell test.

**Table 8:** The Mean and Median Values of Cephalometric Parameters Before and After Treatment.

Parameter	Before Therapy	After Therapy	*p*-Value	Effect Size
SNA (°), mean (SD)	79.38 (2.91)	79.77 (2.95)	0.102 ^a^	0.35 *
SNB (°), mean (SD)	75.61 (3.47)	74.82 (3.41)	0.002 ^a^	0.73 *
ANB (°), median (Q1, Q3)	4.05 (3.35–4.9)	4.8 (3.3–6.12)	<0.001 ^b^	0.48 **
AFH (mm), median (Q1, Q3)	112.85 (109.28–118.72)	115.3 (112.58–118.88)	<0.001 ^b^	0.58 **
PFH (mm), median (Q1, Q3)	72.15 (67.38–78.05)	71.7 (67.35–75.78)	0.864 ^b^	0.02 **
PFH/AFH (%), median (Q1, Q3)	64.17 (61.39–66.1)	63 (59.68–64.51)	0.012 ^b^	0.36 **
Condylar angle (°), mean (SD)	140.84 (8.18)	144.42 (8.87)	0.007 ^a^	0.6 *

^a^ paired *t*-test, ^b^ Wilcoxon’s signed rank test; * Cohen D effect size for paired *t*-test, ** R effect size for Wilcoxon’s signed rank test; SD—standard deviation; Q1—lower quartile; Q3—upper quartile; SNA—Sella-nasion to A point; SNB—Sella-nasion to B point; ANB—A point to B point; AFH—anterior facial height; PFH—posterior facial height.

**Table 9:** Values of Cephalometric Parameters in Unilateral and Bilateral Pathology Groups Before and After Treatment.

	Unilateral Surface Erosion/Subcortical Cyst (11/24)			Bilateral Surface Erosion/Subcortical Cyst (11/24)		
Parameter	Before Therapy	After Therapy	*p*-Value	Before Therapy	After Therapy	*p*-Value
SNA (°)	78.67 (3.08)	79.17 (3.67)	0.239 ^a^	80.29 (2.83)	80.62 (2.19)	0.331 ^a^
SNB (°)	74.20 (72.80–75.80)	73.60 (71.80–75.50)	0.059 ^b^	76.09 (3.46)	75.15 (3.23)	0.002 ^a^
ANB (°)	3.70 (2.10)	4.93 (3.13)	0.028 ^a^	4.10 (3.60–5.2)	5 (4.5–6.5)	0.006 ^b^
AFH (mm)	114.33 (9.33)	117.01 (8.08)	0.007 ^a^	112 (111.40–118.60)	116.2 (112.7–118.7)	0.006 ^b^
PFH (mm)	72.82 (6.51)	72.14 (5.95)	0.435 ^a^	71.84 (6.44)	72.32 (7.09)	0.691 ^a^
PFH/AFH (%)	64.38 (57.99–66.51)	63.44 (56.74–65.59)	0.286 ^b^	63.72 (3.06)	61.65 (2.71)	0.014 ^a^
Condylar angle (°)	142.5 (138.1–147.7)	142.5 (134–152.7)	0.444 ^b^	139.92 (7.32)	144.73 (7.41)	0.003 ^a^

^a^ paired *t*-test, ^b^ Wilcoxon’s signed rank test; SNA—Sella-nasion to A point; SNB—Sella-nasion to B point; ANB—A point to B point; AFH—anterior facial height; PFH—posterior facial height.

**Table 10:** Occlusion Parameters for the Right Canine, Left Canine, and Incisors Before and After Therapy.

Right Canine Before Therapy	Right Canine After Therapy	Left Canine Before Therapy	Left Canine After Therapy	Incisors Overbite Before Therapy	Incisors Overbite After Therapy
1: 11/24 (45.83%) =>	1: 4/24 (36.36%)	1: 12/24 (50%) =>	1: 3/24 (25%)	NOB: 17/24 (70.83%) =>	NOB: 2/24 (11.76%)
	D ½ c: 6/24 (54.55%)		D ½ c: 9/24 (75%)		
	D 1 c: 0/24 (0%)		D 1 c: 0/24 (0%)		NOB: 2/24 (11.76%)
	M ½ c: 1/24 (9.09%)		M ½ c: 0/24 (0%)		DB: 0/24 (0%)
D ½ c: 11/24 (45.83%) =>	1: 0/24 (0%)	D ½ c: 9/24 (37.5%) =>	1: 0/24 (0%)	DB:5/24 (20.83%) =>	NOB: 3/24 (60%)
	D ½ c: 8/24 (72.73%)		D ½ c: 7/24 (77.78%)		
	D 1 c: 3/24 (27.27%)		D 1 c: 1/24 (11.11%)		DB: 1/24 (20%)
	M ½ c: 0/24 (0%)		M ½ c: 1/24 (11.11%)		OB: 1/24 (20%)
D 1 c: 1/24 (4.17%) =>	1: 0/24 (0%)	D 1 c: 3/24 (12.5%) =>	1: 1/24 (33.33%)	OB: 2/24 (8.33%) =>	NOB: 0/24 (0%)
	D ½ c: 1/24 (100%)		D ½ c: 0/24 (0%)		
	D 1 c: 0 (0%)		D 1 c: 0 (0%)		
	M ½ c: 0 (0%)		M ½ c: 2 (66.66%)		DB: 0/24 (0%)
M ½ c: 1/24 (4.17%) =>	1: 1 (100%)	M ½ c: 0/24 (0%) =>	1: 0 (0%)		
	D ½ c: 0 (0%)		D ½ c: 0 (0%)		
	D 1 c: 0 (0%)		D 1 c: 0 (0%)		OB: 2/24 (100%)
	M ½ c: 0 (0%)		M ½ c: 0 (0%)		
*p* = 0.038 ^a^		*p* < 0.001 ^a^		*p* < 0.001 ^a^	

1—dental class I, D one-half c—class II with one-half cusp distalization, D 1 c—class II with one cusp distalization, M one-half c—class III with one-half cusp distalization, NOB—normal overbite, DB—deep bite, OB—open bite, ^a^ Stuart–Maxwell test.

## Data Availability

Data are contained within the article. The data presented in this study are available on request from the corresponding author due to ethical reasons.

## Abbreviations

TMD	Temporomandibular disorders
TMJ	Temporomandibular joint
CBCT	Cone beam computed tomography
SNA	Sella-Nasion-A point
SNB	Sella-Nasion-B point
ANB	A point-Nasion-B point
AFH	Anterior facial height
PFH	Posterior facial height
DC/TMD	Diagnostic Criteria for Temporomandibular Disorders
2D	Two-dimensional
CT	Computed tomography
CI	Confidence interval
CR	Centric relation
RDC/TMD	Research Diagnostic Criteria for Temporomandibular Disorders
3D	Tridimensional
ALARA	As Low As Reasonably Achievable
S	Sella
N	Nasion
Me	Menton
Go	Gonion
Ar	Articulare
ICC	Intra-observer correlation coefficient
1	Class I
D ½ c	Class II with one-half cusp distalization
D 1 c	Class II with one cusp distalization
M ½ c	Class III with one-half cusp mesialization
M 1 c	Class III with one cusp mesialization
NOB	Normal overbite
DB	Deep bite
OB	Open bite
